# Effects and potential mechanisms of luteolin on cisplatin mediated inhibition of cervical adenocarcinoma

**DOI:** 10.1515/biol-2025-1274

**Published:** 2026-05-11

**Authors:** Weixin Zhao, Jian Liu, Shuhua Zhao, Jingying Zheng, Jing Zhao, Taiwei Wang, Mengqi Wang, Junyu Chen, Wenxi Tan

**Affiliations:** Department of Gynaecology and Obstetrics, The Second Hospital, Jilin University, 218 Ziqiang Street, Changchun, Jilin, 130022, China; Department of Gynaecology and Obstetrics, Second Affiliated Hospital, Soochow University, Suzhou, China; Department of Rehabilitation, School of Nursing, Jilin University, Changchun 130022, China; Department of Gynaecology and Obstetrics, Women’s Hospital, Zhejiang University, School of Medicine, 368 Jiren Street, Hangzhou, Zhejiang, 311215, China

**Keywords:** *Brucea javanica*, cervical adenocarcinoma, network analysis, luteolin, cisplatin, chemosensitization

## Abstract

*Brucea javanica* (BJ) is widely used as an adjuvant therapy for malignant tumours in China. Currently, scholars at home and abroad mainly focus on the nonpolar-constituent fatty acids, which are the common main constituents of dietary edible oil, while the characteristic polar constituents such as flavonoid glycosides have not been explored. Luteolin (LU), as an active polar constituent of BJ, has been proven to be a platinum sensitizer in many cancers. The study employed a combination of network pharmacology, *in vitro* cell assays (HeLa, SiHa), and *in vivo* xenograft models to demonstrate that LU can potentiate the efficacy of CDDP while reducing its dosage and toxicity by inducing apoptosis, G2/M cell cycle arrest, and inhibition of migration/invasion. Mechanistically, LU appears to act through PI3K/AKT signaling pathway. This offers a highly promising adjuvant therapeutic strategy to improve treatment tolerance and prolong survival in cervical cancer (CC) patients.

## 1 Introduction

Cervical cancer (CC) remains the most prevalent malignant tumour of the female reproductive system in the world, with high recurrence and mortality rates, but it is also a serious economic and social burden [1]. Cisplatin (CDDP) is the standard first-line agent for chemotherapy in the National Comprehensive Cancer Network (NCCN) of CC [2], 3]. Long-term conventional chemotherapy with a relatively high dose will not only lead to a decrease in the chemotherapy effect of tumour cells and tolerance of the body but also make the decrease in tumour markers not obvious. The dosage can be appropriately increased according to the body surface area and creatinine value, but the increase in side effects will lead to the interruption of clinical treatment, such as anaemia, neutropenia, impaired liver function, impaired kidney function, and severe nausea and vomiting. That is, due to intolerance and organ toxicity, the efficacy of cisplatin is impaired [4]. Therefore, adjuvants that not only improve the efficacy of CDDP but also reduce the dose and toxicity of CDDP, thus prolonging the survival of cervical cancer patients, are very desirable.

*Brucea javanica* (L.) Merr. [Simaroubaceae] (Chinese name Yadanzi) is a plant belonging to the plant genus *Brucea* of the Simaroubaceae family [5], which is used as medicine with its fruits and leaves [6]. Luteolin (LU), as an active ingredient of BJ, has been proven to be a platinum sensitizer in many cancers [7], 8]. Although LU exhibits broad anticancer potential, most studies have prioritized prevalent or aggressive cancers (e.g., lung, breast, ovarian) [9]. While previous studies have reported anti-cancer effects of luteolin in cervical cancer models, its synergistic potential with cisplatin and the underlying mechanisms remain incompletely understood. This study aimed to investigate the combined effect of luteolin and cisplatin on cervical cancer cells and *in vivo*.

Network analysis integrates systems bioinformatics and pharmacology to identify key active compounds in traditional Chinese medicine (TCM) [10], 11]. By mapping drug-component-disease-action target interactions, it reveals molecular-level synergies and visualizes relationships between genes, proteins, and diseases, advancing TCM research and development [12].

To explore and prove the therapeutic potential of LU for CC, in this study, we used the network analysis method for the first time and found that LU, as an effective component of BJ, could be used as an adjuvant therapy to chemotherapy against CC and predicted its possible mechanism of action. Meanwhile, *in vitro* HeLa cells and *in vivo* xenograft tumour nude mouse models were established to validate the potential of LU as a CDDP sensitizer predicted by network analysis.

## 2 Materials and methods

Data in network pharmacology analysis were retrieved up to October 2021. The Second Hospital of Jilin University Medical Ethics Committee (Changchun, China, no.2023055) authorized protocols. The *in vivo* experiments were conducted in Changchun, China, in June 2022. The experimental procedures were approved by Jilin University Experimental Bioethics Committee (Changchun, China, no.202073). Animal experiments complied with the arrival guidelines and the National Institutes of Health Guidelines for the Care and Use of Laboratory Animals.

**Ethical approval:** The research related to animal use has been complied with all the relevant national regulations and institutional policies for the care and use of animals, and has been approved by the Jilin University Experimental Bioethics Committee (Changchun, China, no.202073).

### 2.1 Collection and screening of bioactive ingredients in BJ

“BJ” was input into the Traditional Chinese Medicine Systems Pharmacology database and Analysis Platform database (TCMSP, https://old.tcmsp-e.com/tcmsp.php) for query, and the chemical constituents of BJ were sorted out. Meanwhile, ADME screening including human oral bioavailability (OB) > 30 % and drug-like quality (DL) > 0.18, was implemented for filtration of the collected ingredients [13]. OB is a measure of the speed and extent of drug absorption into blood circulation, and DL refers to the similarities with known drugs [14]. Targets for each active ingredient predicted by TCMSP were collected separately and tabulated. The UniProt database (https://www.uniprot.org/uploadlists) was used to standardize these targets [15].

### 2.2 Tabulate the disease-target-gene data set

With “CC” as the key word and the species limited to “*Homo sapiens*” in GeneCards (https://www.genecards.org), annotation and prediction were carried out, and the genes related to “CC” were obtained [13]. Genes with a correlation greater than one point were collected as “Disease-target-gene”.

### 2.3 Venn the intersection gene

The intersection target genes of “CC” and “BJ” were drawn by the Venny 2.1.0 mapping platform (https://bioinfogp.cnb.csic.es/tools/venny/index.html) [15]. The results that were irrelevant to CC were manually deleted, and the remaining names were used to draw the VENN map of the target gene where the disease and the drug intersect.

### 2.4 Visualize the “M-C-T-P” regulatory network

The “Medicine-compounds-targets-pathways” (M-C-T-P) network was visualized through Cytoscape_3.7.2 (https://cytoscape.org). The more lines were connected, the more genes were regulated by the component, which means that it is the prior bioactive compound to anti-CC in BJ.

### 2.5 Protein-protein interaction (PPI) network construction

The main active ingredients of CC target genes were imported into the STRING 11.0 database (https://cn.string-db.org) for analysis with mode set to “Multiple proteins”, and the species was limited to “*H. sapiens*”. After prereading the data, the remaining proteins had a reliability ≥ 0.99, and the isolated proteins were hidden. Then, a protein-protein interaction (PPI) network was constructed with a medium confidence interaction score threshold of 0.4, and targets that did not meet this criterion were removed. The barplot plotting function was used to draw a histogram sorted by the number of connected nodes of proteins to find core genes.

### 2.6 GO and KEGG enrichment analyses

Gene Ontology (GO) enrichment analysis and Fisher exact test were performed by R 3.6.0 software to determine which functions the genes were enriched in, from molecular function of targets, cellular components and involved biological processes [16]. In addition, the *P*-value was positively associated with the degree of enrichment. The result was drawn into corresponding histograms and bubble charts with *R*. The enrichment results with *P* < 0.05 after screening and correction were selected. Kyoto Encyclopedia of Genes and Genomes (KEGG) pathway enrichment analysis was employed to determine which pathways were acted upon by the hub targets. To visualize the data, the target-pathway intervention mechanism diagrams were depicted by *R* software with the Bioconductor package loaded. Target-path mechanism analysis of the pathway in the KEGG table was performed, and the intervened target is marked in red.

### 2.7 Cell culture

The human cervical cancer HeLa and Siha cell lines were obtained from Procell Life Science & Technology Co., Ltd. The cells were cultured in Iscove’s modified Dulbecco’s medium (IMDM) supplemented with 10 % foetal bovine serum (FBS) and 1 % penicillin-streptomycin, cultivated in a 37 °C incubator containing 5 % CO_2_.

### 2.8 Cell viability experiment

In short, HeLa and Siha cells during the logarithmic growth phase were digested and prepared into a single-cell suspension, seeded in a 96-well plate at a density of 2,000 cells per well, in a volume of 100 µL, with vibrations added to prevent the cells from settling to the bottom. After 24 h, the medium was removed, and the cells were treated individually or in combination with different concentrations of LU (0, 0.1, 0.5, 5, 10, 20, 40 µM) and CDDP (0, 0.5, 2.5 μg/mL) for 24 and 48 h. Equal volumes of dimethyl sulfoxide (DMSO) were added to the control group. After treatment, 10 µL of Cell Counting Kit-8 reagent (CCK-8) was added to each well, and the incubation was continued for 1 h at 37 °C. Finally, the absorbance value of each well was detected with a microplate reader at 450 nm. The growth inhibition rate in the CCK-8 assay was calculated using the following standard formula:

$$\text{Inhibition Rate}\left(\%\right)=\left[1-\frac{\left({\text{OD}}_{\text{drug}}-{\text{OD}}_{\text{blank}}\right)}{\left({\text{OD}}_{\text{Control}}-{\text{OD}}_{\text{blank}}\right)}\right]\times 100$$

### 2.9 Colony formation assay

The ability of cells to form clones was detected by colony formation assay. After seeding 1,500 HeLa cells in each dish 10 cm in diameter and incubating for 24 h, the culture medium was discarded. Luteolin (0, 5, 10 μM) and CDDP (0, 0.5 μg/mL) were administered separately or in combination for 14 days of incubation until visible colonies appeared. Colonies fixed with 4 % paraformaldehyde were incubated with 10 % Giemsa dye solution for 10 min, images were taken by a camera, and the number of visible colonies was counted.

### 2.10 Flow cytometry analysis of apoptosis and the cell cycle

Cells were seeded in 6-well plates at 1.5 × 10^5^ cells per well and treated at a concentration consistent with the colony formation assay for 24 h. After being harvested, cells were fixed overnight in 70 % precooled ethanol at −20 °C. Fixed cells were then suspended in PBS containing 50 μg/mL propidium iodide (PI), 100 μg/mL RNase-A and 0.2 % Triton X-100 for 30 min at room temperature in the dark. Stained cells were analysed by FACSCAN flow cytometry (BD, San Jose, CA, USA), and the percentage of cells in each phase was analysed by Modfit LT five software.

For apoptosis detection, after harvesting the cells in the same way as in the flow cytometry cycle assay, cells were stained with 5 µl of Annexin V-FITC and 5 µL of PI for 15 min at room temperature in the dark. Machine and analytical methods were consistent with the cell cycle described above.

### 2.11 Hoechst staining

Briefly, cells were seeded in 6-well plates at 1.5 × 10^5^ cells per well and treated at a concentration consistent with the colony formation assay for 24 h. After being harvested and washed with PBS twice, cells were fixed with 4 % paraformaldehyde in PBS for 10 min, followed by staining with Hoechst 33,342 reagent for 10 min at room temperature. Finally, the cells were observed under a fluorescence microscope and images were taken with a digital camera (BX51, Olympus, Japan).

### 2.12 Wound-healing assay

Briefly, cells were seeded into a 6-well plate at 1.5 × 10^5^ cells per well and cultured in complete growth medium at 37 °C in a 5 % CO_2_ incubator until they reached approximately 80 % confluence. A uniform straight scratch was then created in the monolayer of each well using a sterile 200 µL pipette tip, guided by a ruler placed beneath the plate to ensure consistency. The dislodged cells and debris were gently removed by washing twice with PBS. Subsequently, the cells were incubated in low-serum medium (containing 1 % FBS) to minimize the influence of cell proliferation on wound closure. Treatment with LU, CDDP, or their combination was administered at concentrations consistent with those used in the colony formation assay. An equal volume of vehicle (0.05 % DMSO) was added to the control wells. Images of the wound at the same marked location were captured at 0 h (immediately after washing) and 24 h post-scratch using an optical microscope. The migratory ability was quantified by measuring the residual wound area at 24 h relative to the initial wound area at 0 h using ImageJ software, and the percentage of wound closure was calculated.

### 2.13 Transwell invasion assay

Six groups of cells were treated for 24 h and then prepared into a suspension with serum-free media. A total of 2 × 10^4^ cells were seeded into the upper chamber of a Transwell containing 100 µL of serum-free medium in total. The lower chamber contained 600 µL of medium containing 10 % foetal bovine serum (FBS). After 24 h of treatment, the chambers were removed and stained with Wright-Giemsa staining solution and processed as described for the colony formation experiments above. Images were taken with a light microscope.

### 2.14 Western blot analysis

Cold RIPA lysis buffer premixed with protease-phosphatase inhibitor was used to extract soluble protein samples from six groups of experimental cells. Protein concentrations were determined using a Coomassie Plus (Bradford) Assay Kit. A total of 15 µg of protein was loaded per lane for electrophoresis and transferred onto polyvinylidene fluoride (PVDF) membranes. The membrane was bolcked with 5 % skim milk for 1 h at room temperature, followed by incubation with primary antibodies, including anti-cyclin-dependent kinase 4 (CDK4), anti-Cyclin B1 and Glyceraldehyde-3-phosphate dehydrogenase (GAPDH) antibody on a shaker at 4 °C overnight. Subsequently, following incubation with secondary- HRP antibodies at room temperature for 1 h, and the levels of protein expression were detected by an electrochemiluminescence (ECL) plus kit. GAPDH was used as an internal control for normalizing band intensities. During ECL luminescence detection, the initial exposure time ranged from 30 s to 5 min and was dynamically adjusted based on signal strength. All experiments were performed with three independent biological replicates. An imaging densitometer and its accompanying Gel Analysis software (Clinx Science Instruments Co., Ltd.) were used to perform semiquantitative measurements of the densities of the specific bands.

### 2.15 In vivo experiments

Twenty-four female BALB/c nu/nu mice (5 weeks old, 16–20 g) were obtained from Beijing Vital River Laboratory Animal Technology Co., Ltd., housed under specific pathogen-free (SPF) conditions at 22 °C and 50–60 % humidity with an alternating 12 h light/dark cycle and provided with food and water *ad libitum*. After being acclimated for one week before the experiment, HeLa cells (2 × 10^6^ cells) in a volume of 100 μl of PBS were injected subcutaneously into the right dorsal side of athymic mice. After 2 weeks of inoculation, tumours were visible with a volume of approximately 80–100 mm^3^. The mice were randomly grouped (6 mice per group) as follows: i) Control group: DMSO 0.5 ml/kg and *β*-cyclodextrin 6 mg/kg; ii) LU group: 20 mg/kg; iii) CDDP group: 3 mg/kg; and iiii) LU combined with CDDP group: received doses of each drug consistent with the monotherapy group. CDDP was dissolved in PBS before use and the injection volume was 100 μl, and LU was dissolved in 1 % DMSO and 20 % *β*-cyclodextrin. All reagents were injected intraperitoneally into mice every other day. The mice in the control group received equal doses and frequencies of DMSO and *β*-cyclodextrin as the LU group.

Tumour size, as well as the weight of each mouse, was recorded every other day, and tumour size was measured and recorded as *volume* = *length* × *width* ^2^/2. Tumor growth rates were assessed by plotting tumor volume over time for each experimental group. Specific humane endpoints for euthanasia included a tumor volume exceeding 2,000 cubic millimeters, ulceration, a tumour diameter greater than 20 mm, or significant weight loss greater than 20 %. Death was confirmed through the observation of respiratory arrest, cardiac arrest, and loss of reflexes. All mice were studied throughout the full experiment. After 16 days of treatment, the mice were euthanized via cervical dislocation following an overdose of isoflurane. Following the induction of anesthesia, isoflurane was removed upon confirming complete muscle relaxation and loss of eyelid reflexes. Subsequently, mice were euthanized by cervical dislocation, and tumours were harvested for subsequent analysis of volume and weight. Organs, including the heart, liver, spleen, lungs, and kidneys, were collected for further analysis. Whole blood samples were obtained from the retro-orbital venous sinus of each mouse.

### 2.16 TUNEL assay

Deparaffinized sections of mouse tumour tissues were stained with a TUNEL assay kit. Then, the apoptotic cells on the slides were observed under a fluorescence microscope, and images were taken with a digital camera (BX51, Olympus, Japan).

### 2.17 Histologic analysis

Histologic analysis was conducted as previously described [17]. After 10 % formalin fixation, the tumour, heart, liver, kidney, and lung specimens of rats were embedded in paraffin, and then 4-µm-thick tissue sections were stained with haematoxylin and eosin (H&E). Sections of tumour tissue were incubated with primary antibodies overnight at 4 °C, including anti-p-PI3K, anti-*p*-AKT, anti-*p*-ERK, anti-cyclin-B1, anti-BAX, anti-cleaved-caspase-3 (c-caspase-3) and anti-MMP2. Then, the sections were incubated with the secondary antibody which is goat anti-rabbit IgG conjugated with horseradish peroxidase, at 37 °C for 30 min. The sections were developed with a DAB horseradish peroxidase chromogenic kit. Five fields of view were randomly selected under an optical microscope (Motic Incorporation, Ltd.) at ×200 magnification for each slice, and their average value was taken to calculate the positive ratio.

### 2.18 Blood examination

The liver function index, renal function index and routine blood index of mice were analysed by a Shinova haematology analyser, including alanine transaminase (ALT) count, aspartate aminotransferase (AST) count, albumin (ALB) count, *γ*-glutamyl transpeptidase (*γ*-GT) count, total bile acid (T-BIL) count, blood urea nitrogen (BUN) count, creatinine (CREA) count, uric acid (UA) count, white blood cell (WBC) count, red blood cell (RBC) count, haemoglobin (HGB) count and blood platelet (PLT) count.

### 2.19 Statistical analysis

Image-Pro Plus 6.0 software (Media Cybernetics, Inc.) was used to analyze the mean integrated optical density (IOD) of the TUNEL and immunohistochemistry results. Specifically, five randomly selected high-power fields (400 × magnification) per sample were captured with consistent threshold settings applied across all samples. Positive signals were identified and quantified. The normality of data distribution was verified using the Shapiro-Wilk test, and homogeneity of variance was assessed via Levene’s test. Based on these results, parametric tests were selected. SPSS (version 26; IBM Corp.) software was used to perform one-way ANOVA followed by Bonferroni’s post hoc test for comparison between multiple groups. Quantitative data were collected from three independent experimental repeats *n* = 3 and are presented as mean ± SD to reflect the variability within the dataset. GraphPad Prism 8.0.1 was used to visualize the results, and statistical significance was defined as meaningful at *P* < 0.05.

## 3 Results

### 3.1 Candidate compound screening for BJ

A total of 67 BJ bioactive ingredients were obtained from the TCMSP computer system, 15 of which were left after being screened by ADME (OB > 30 % and DL > 0.18) and are presented in Table 1. Eighty related targets of the effective ingredients of BJ were collected with TCMSP.

**Table 1:** The 15 chemical components obtained by *Brucea javanica* screening.

Mol id	Molecule name	OB (%)	DL
MOL008073	brusatol	45.69	0.75
MOL000358	beta-sitosterol	36.91	0.75
MOL008077	yadanzioside B	46.16	0.31
MOL000006	luteolin	36.16	0.25
MOL008068	bruceoside A_qt	31.05	0.75
MOL008089	yadanzioside H	62.77	0.32
MOL008091	yadanzioside I	61.13	0.38
MOL008093	yadanzioside J	38.7	0.30
MOL008097	yadanzioside L	31.37	0.27
MOL008099	yadanzioside M	45.04	0.23
MOL008105	yadanzioside P	58.76	0.29
MOL008108	yadanziolide C_qt	31.8	0.66
MOL008109	yadanziolide D	55.76	0.65
MOL008110	bruceoside B	56.54	0.32
MOL008112	bruceine C	31.38	0.66

### 3.2 The CC-target-gene data set and the venn diagram

**Figure 1:**
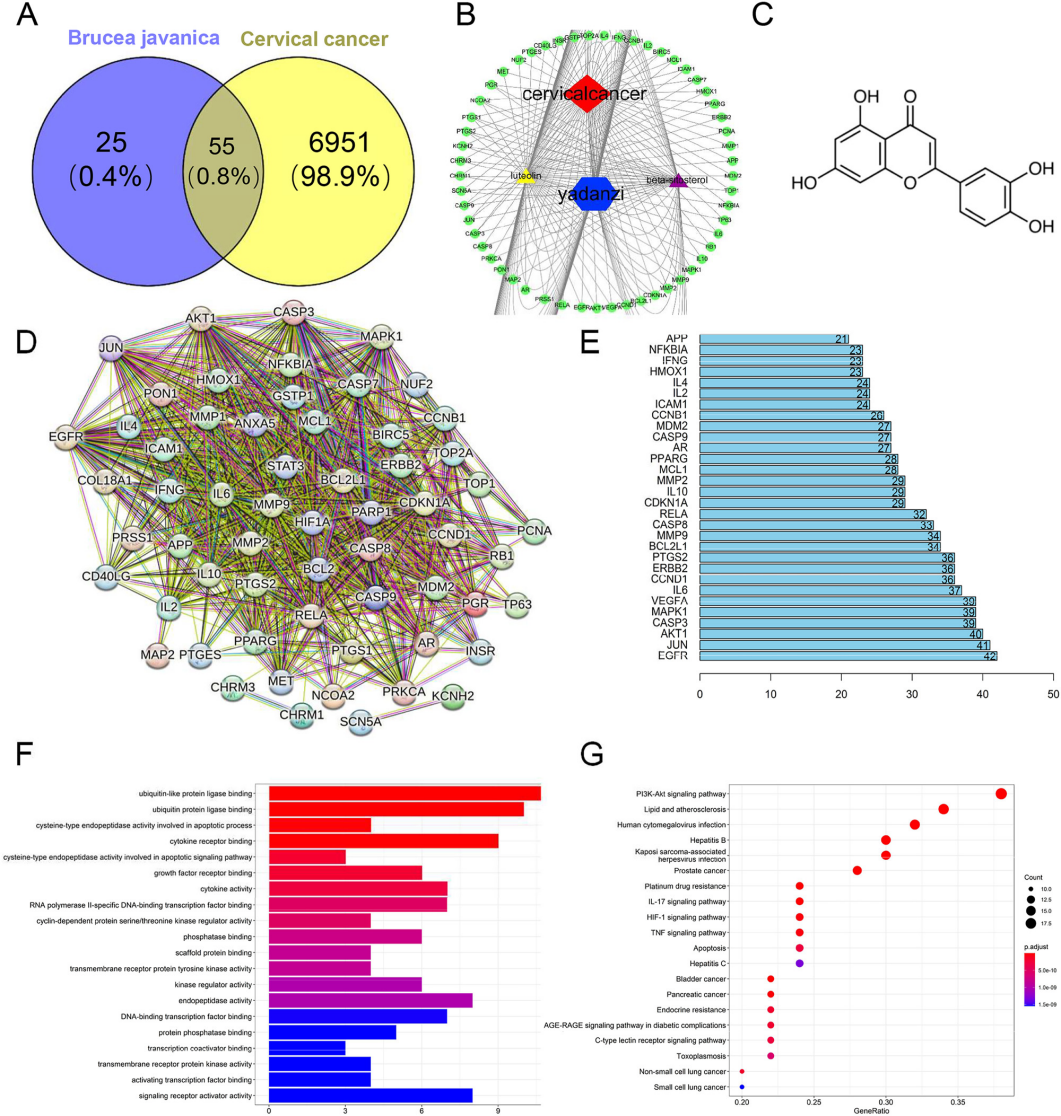
Network analysis of *Brucea javanica*. A. Venn diagram of the number of potential targets for *Brucea javanica* in the treatment of cervical cancer. B. The “M-C-T-P” network with cytoscape. The green box node represents the target gene, the purple square node represents the compound component, and the grey line suggests the interaction between the nodes. C. The structure of luteolin (LU). D. Protein-protein interaction network of potential cervical cancer targets of luteolin. The round ball at the node represents the target protein. The lines between the spheres represent the interaction between nodes. E. The nodes are sorted by the number of connections, and the top 30 are represented. F and G. GO analysis presents the enriched proteins, and KEGG analysis presents the therapeutic mechanism of BJ’s active ingredients against CC.

A total of 7,006 different gene symbols related to CC were retrieved from GeneCards, which is a human gene database. The 55 BJ-CC overlapping genes were mapped with Venny 2.1.0, including AKT1, JUN, mitogen-activated protein kinase 1 (MAPK1), matrix metallopeptidase 9 (MMP9), epidermal growth factor receptor (EGFR), caspase (CASP) 3, vascular endothelial growth factor A (VEGFA), interleukin (IL)-6, and BCL2 (Figure 1A).

### 3.3 Construction of the M-C-T-P network with cytoscape

The “medicine-compound-target-pathway” (M-C-T-P) network was visualized through Cytoscape-3.7.2 in Figure 1B. Fifty-eight nodes and 126 connections were included in the network. The green box node represents the target gene, the purple square node represents the compound component, and the grey line suggests the interaction between the nodes. There are complex interactions between bioactive anti-CC compounds and target genes. A botanical drug ingredient had multiple disease-targeting genes, and the target genes were associated with more than one ingredient. For CC, luteolin acted on 46 target genes and *β*-sitosterol acted on 23. The more connected nodes there are, the more genes were regulated by the component. The structure of luteolin was shown in Figure 1C. In addition, they acted on the same gene at the same time, which suggested that there was synergy between the compounds.

### 3.4 PPI network construction

Protein genes did not work independently, but were interconnected to function at multiple levels. Fifty-five target genes of CC-BJ were entered into STRING to construct a protein interaction network (PPI) in Figure 1D, which included relationships other than just coexpression, gene fusion and neighbourhood. The round ball at the node represents the target protein. The lines between the spheres represent the interaction between nodes. A total of 55 nodes and 586 edges were collected. Based on the number of neighbours of the node genes, the top 30 targets were calculated and counted by Barplot (Figure 1E). EGFR (42), JUN (41), AKT1 (40), CASP3(39) and MAPK1 (39) were the top five nodes. Twenty-nine of 30 were targeted by luteolin to exert anticancer effects, yet only five out of 30 were targeted by *β*-sitosterol. The obtained results suggested that luteolin may be the most effective and most functional of all candidate ingredients used by BJ to fight CC.

### 3.5 GO and KEGG enrichment

To explore the functions that the proteins were enriched in, GO analysis was performed by R software loaded with human Bioconductor packages. The top 20 with the lowest *P*-values of the 65 results are shown in Figure 1F. The redder is the colour, the lower is the *P*-value. The obtained results revealed that the functions of proteins encoded by the BJ-CC target genes were abundant in the biological processes of apoptosis, autophagy, negative regulation of gene expression, protein phosphorylation and molecular functions such as protein binding and transcription factor binding. To reveal the therapeutic mechanism of BJ’s active ingredients against CC, a total of 96 pathways with significant differences (*P* < 0.05) were analysed by KEGG enrichment analysis. The PI3K-Akt signalling pathway (hsa04151) and platinum drug resistance (hsa01524) signalling pathways ranked in front of 20 pathways with high likelihood associated with BJ against CC (Figure 1G).

### 3.6 Cell viability assay

**Figure 2:**
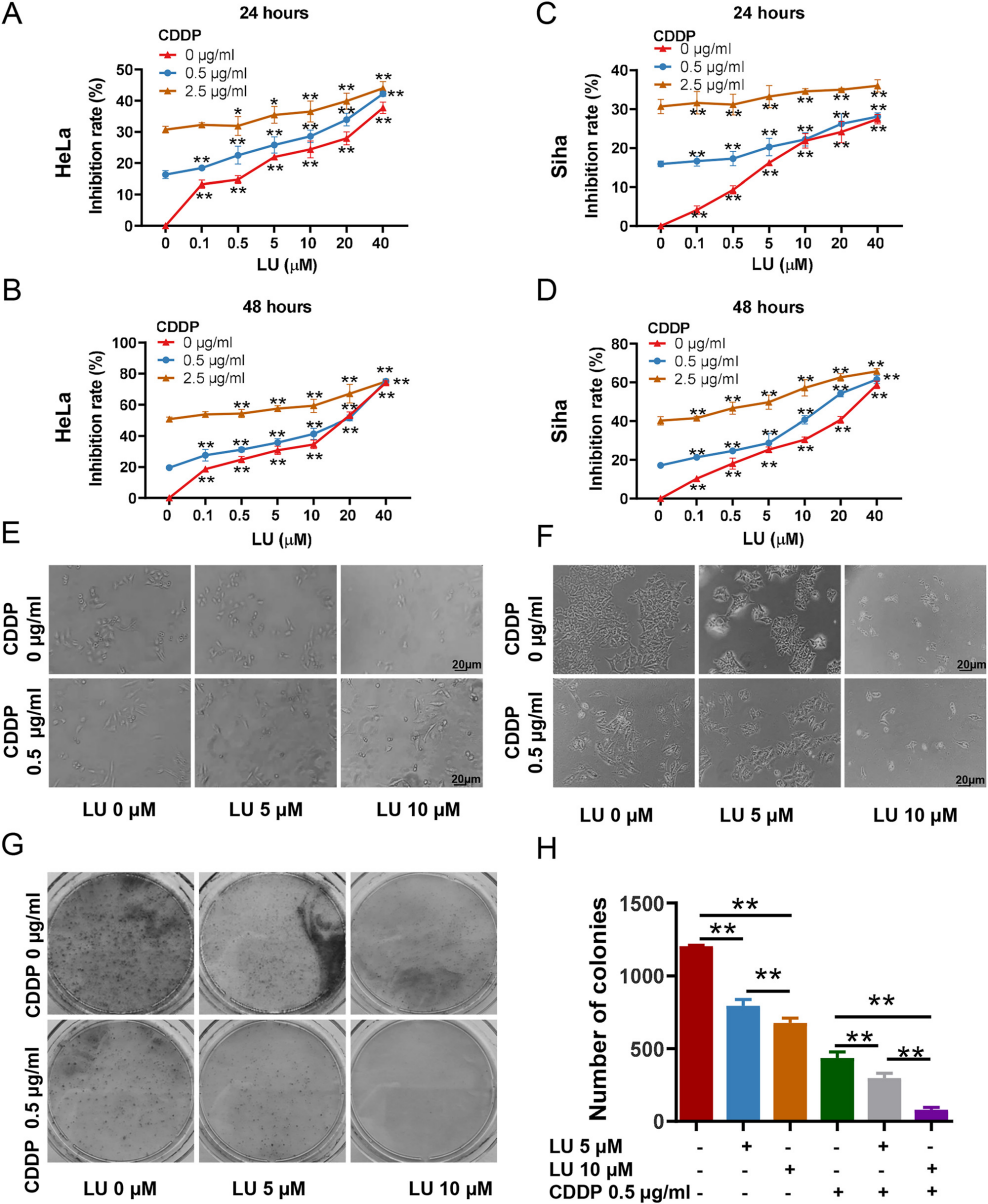
Luteolin inhibits the proliferation and colonization capacity of HeLa cells. HeLa cells were treated with different concentrations of LU (0, 0.1, 0.5, 5, 10, 20, 40 µM 0 μM) and CDDP (0, 0.5, 2.5 μg/mL) for 24 h and 48 h. A and B. HeLa cell proliferation induced by different concentrations of LU and CDDP for 24 h and 48 h was detected by CCK8 assay. C and D. Siha cell proliferation induced by different concentrations of LU and CDDP for 24 h and 48 h was detected by CCK8 assay. E and F. The number and morphology of hela and siha cells were observed with microscope. G and H. HeLa cell proliferation was detected by colony formation assay and statistical analysis. *P < 0.05, **P < 0.01 compared with the control group.

First, the CCK-8 assay results showed that the growth inhibitory effect of luteolin on HeLa and Siha cells was dose dependent, and a low dose of 5 μM significantly inhibited HeLa and Siha cells (Figure 2A–C). In the combined group, both 0.5 μg/mL and 2.5 μg/mL CDDP had higher inhibition rates of HeLa and Siha cells by LU, and the sensitization effect of the former was the most obvious. Therefore, 0.5 μg/mL CDDP was selected to evaluate the synergistic inhibitory effect of LU on HeLa and Siha cells. In addition, the inhibition rate of HeLa and Siha cells treated in the combination group at 24 h (Figure 2A–C) and 48 h (Figure 2B–D) was significantly higher than that in the CDDP group (*P* < 0.05). The number and morphology of Hela and Siha cells were observed with microscope (Figure 2E and F). Colony formation experiments were performed to verify the effect of LU and CDDP on HeLa and Siha cell proliferation (Figure 2G). As LU dosage increased, the number of cell clones was significantly reduced (*P* < 0.05), and the colonization capacity of HeLa and Siha cells exposed to combination therapy was significantly inhibited (Figure 2H) after 14 days of culture compared to the LU or CDDP groups (*P* < 0.05). The obtained results showed that LU could slightly increase Siha cell sensitivity to platinum, which is in line with the available study that Siha cells are more resistant to cisplatin-induced apoptosis than HeLa cells under similar conditions of cisplatin treatment [18].

### 3.7 Cell cycle distribution

**Figure 3:**
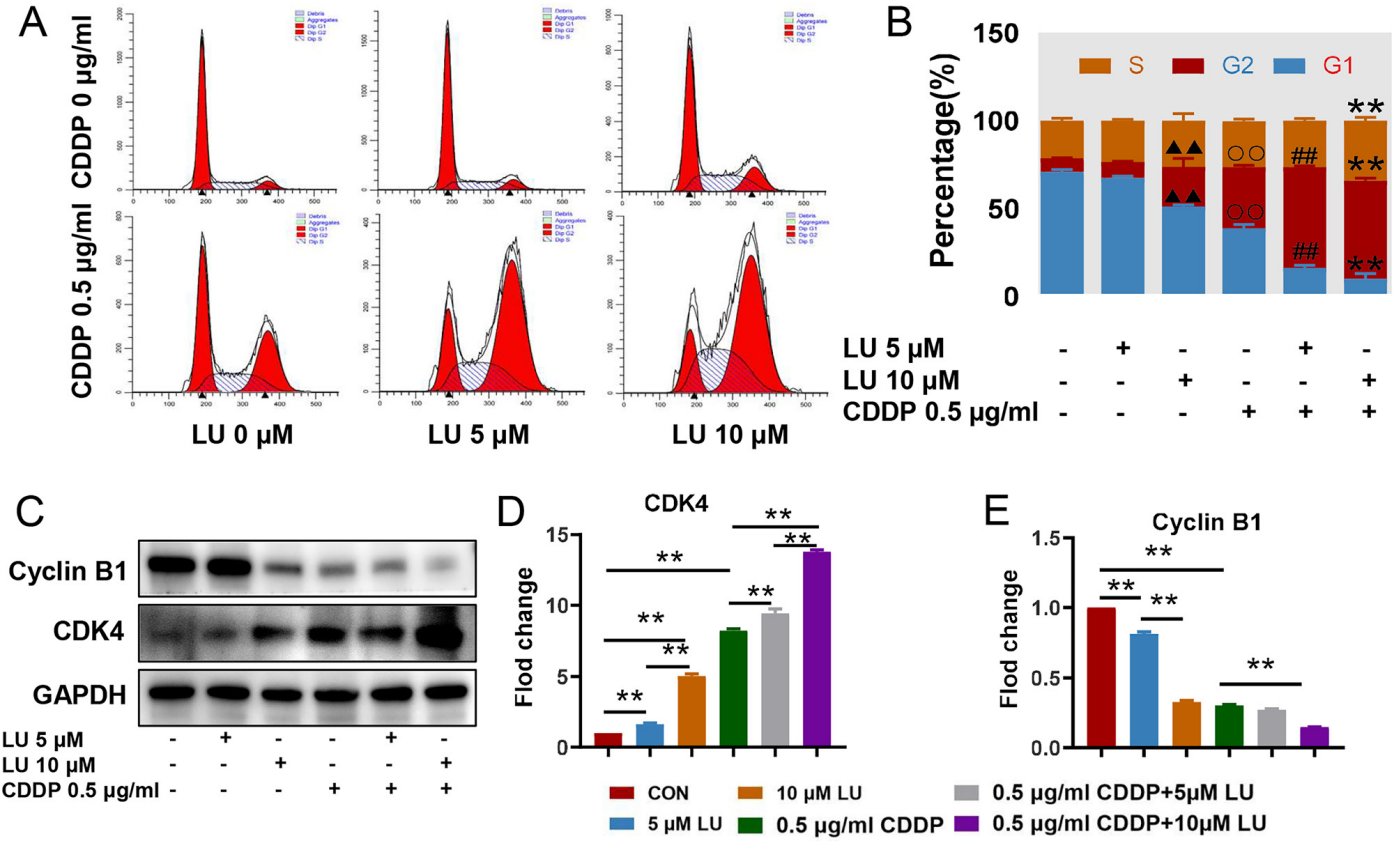
The effect of LU synergizes with CDDP on the HeLa cell cycle. HeLa cells were treated with different concentrations of LU (0, 0.1, 0.5, 5, 10, 20, 40 µM 0 μM) and CDDP (0, 0.5, 2.5 μg/mL). A. The cell cycle of HeLa cells was detected by flow cytometry after treatment with LU and CDDP. B. The percentages of G1, S and G2 phases of the HeLa cell cycle are shown as a column diagram. C, D and E. The expression levels of cyclin B1 and CDK4 in HeLa cells were detected by western blot. The relative protein level was calculated as the ratio of the targeted protein cyclin B1 and CDK4/GAPDH. The data shown are the averages of three experiments ± standard errors of the means. *P < 0.05, **P < 0.01 compared with control cells. #*P* < 0.05, ##*P* < 0.01 compared with control cells. ▲*P* < 0.05, ▲▲*P* < 0.01 compared with control cells. 〇*P* < 0.05, 〇〇*P* < 0.01 compared with control cells.

For the results of the cell cycle analysis, after 0, 5 and 10 μM LU treatment, the percentage of HeLa cells in G2/M phase increased from 7.8 % to 9.0 % and 22.7 %, while that of G1/G0 decreased from 70.8 % to 67.3 % and 51.0 %, respectively. Compared with the CDDP group, the percentage of HeLa cells in the G2/M phase increased from 34.7 % to 55.9 %, while the G1/G0 phase decreased from 38.6 % to 9.8 % after combined treatment with 10 μM LU (Figure 3A). The percentages of G1, S and G2 phases of the HeLa cell cycle are shown as a column diagram in Figure 3B. To explore the mechanism of LU and CDDP on G2/M phase arrest, the expression levels of cell cycle regulatory proteins were detected by western blotting analysis. In this study, the expression level of cyclin B1 in each group was correlated with the proportion of cells in G2 phase, and the higher was the proportion of cells in G2 phase, the lower was the expression of cyclin B1. Compared with that in the CDDP group, the expression level of cyclin B1 in the combination group was significantly decreased (*P* < 0.05, Figure 3C–E). In addition, western blot analysis also showed a decrease in CDK4 protein expression associated with cell arrest in G1/G0 phase (Figure 3C and D).

### 3.8 Analysis of apoptosis

**Figure 4:**
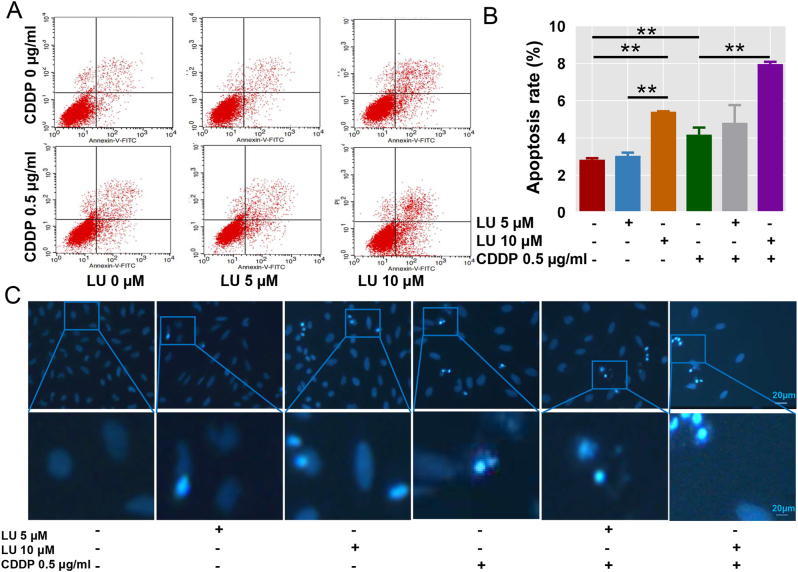
CDDP-induced HeLa cell apoptosis was significantly enhanced by LU. HeLa cells were treated with different concentrations of LU (0, 0.1, 0.5, 5, 10, 20, 40 µM 0 μM) and CDDP (0, 0.5, 2.5 μg/mL). A. The cells were stained with Annexin V- FITC/PI. Cell apoptosis was detected by flow cytometry. The PI + Annexin V+ cells were apoptotic cells. B. The percentages of apoptotic HeLa cells in the cell cycle are shown as a column diagram. C. HeLa cells were stained with hoechst and observed with a microscope. The data shown are the averages of three experiments ± standard errors of the means. *P < 0.05, **P < 0.01compared with control cells.

Regarding the results of flow cytometry apoptosis analysis, after treatment with 0, 5 and 10 μM LU, the late apoptosis rate of HeLa cells was markedly increased from 2.83 % ± 0.09 (mean ± SD) to 3.04 % ± 0.18 and 7.41 % ± 0.03. Compared with the CDDP group, the late apoptosis rate of HeLa cells increased from 6.17 % ± 0.39–7.97 % ± 0.12 after 10 μM LU combined treatment (Figure 4A and B).

For the Hoechst assay, luteolin led to an increase in apoptotic cells compared with the control group, and the combined group resulted in significantly more nuclear shrinkage or fragmentation of apoptotic cells than the CDDP group (Figure 4C).

### 3.9 Wound-healing assay

**Figure 5:**
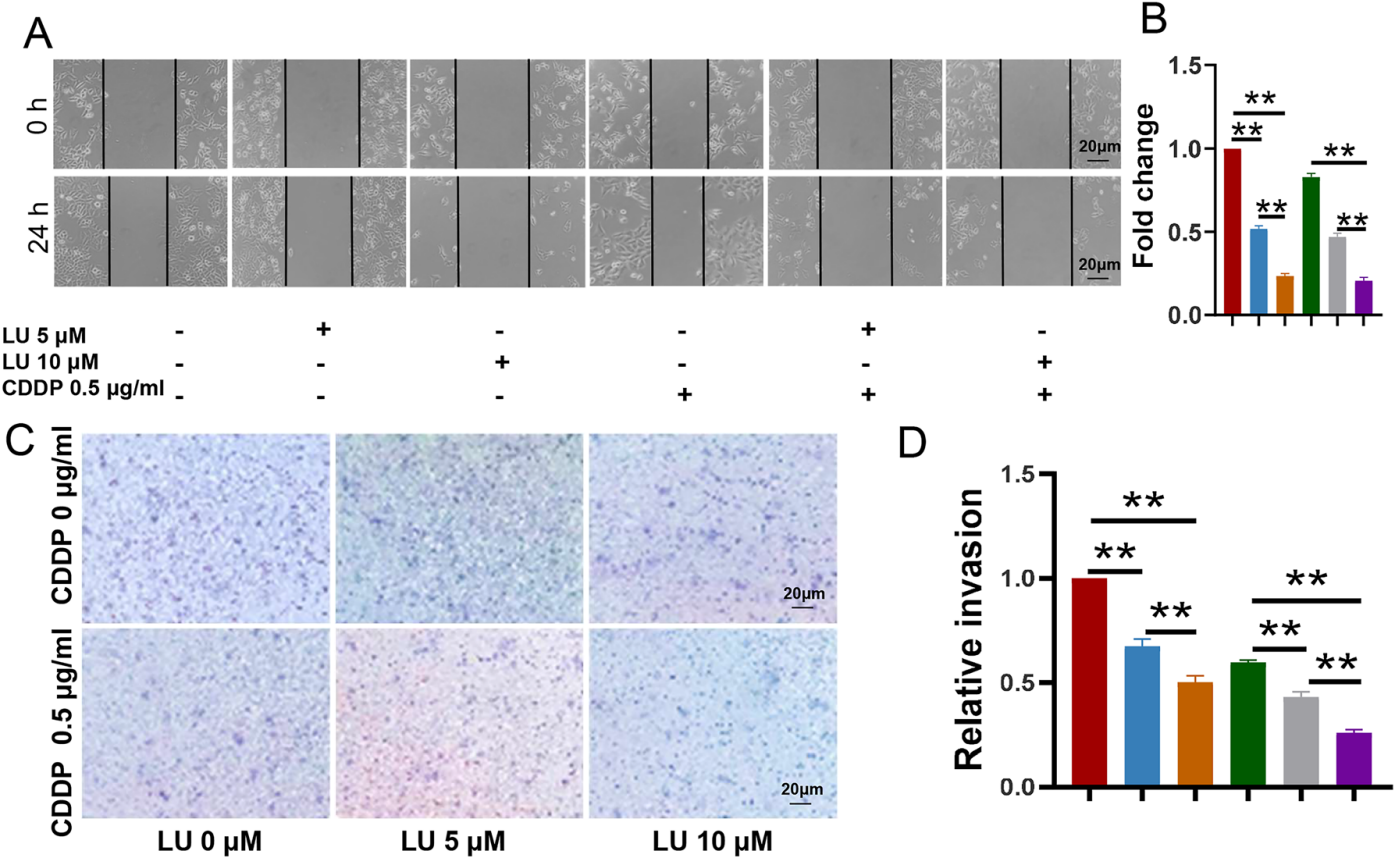
CDDP-inhibited metastasis capabilities of HeLa cells were significantly enhanced by LU. HeLa cells were treated with different concentrations of LU (0, 0.1, 0.5, 5, 10, 20, 40 µM 0 μM) and CDDP (0, 0.5, 2.5 μg/mL). A and B. Cell migration was detected by wound-healing assay. C and D. Cell invasion was detected by transwell assay. The data shown are the averages of three experiments ± standard errors of the means. *P < 0.05, **P < 0.01 compared with control cells. Each colored line represents a different treatment group: Red (control), blue (LU 5 μM), orange (LU 10 μM), green (CDDP 0.5 μg/ml), grey (LU 5 μM + CDDP 0.5 μg/ml) and purple (LU 10 μM + CDDP 0.5 μg/ml). Data are presented as mean ± SD.

After 24 h, the migration rate of HeLa cells decreased from 26.2 % ± 0.92–13.5 % ± 0.23 and 6.1 % ± 0.26 after treatment with 0, 5 and 10 μM LU, respectively. Compared with the CDDP group, the migration rate of HeLa cells in the combined treatment group with 10 μM LU was further reduced from 21.7 % ± 0.83–5.37 % ± 0.2 (Figure 5A and B), and the spacing of the scratches was also significantly wider.

In the Transwell assay, compared with the CDDP group, a significant reduction (*P* < 0.05) in the number of cells cotreated with CDDP and LU invading through the polycarbonate membrane was observed (Figure 5C and D).

### 3.10 *In vivo* experiment

**Figure 6:**
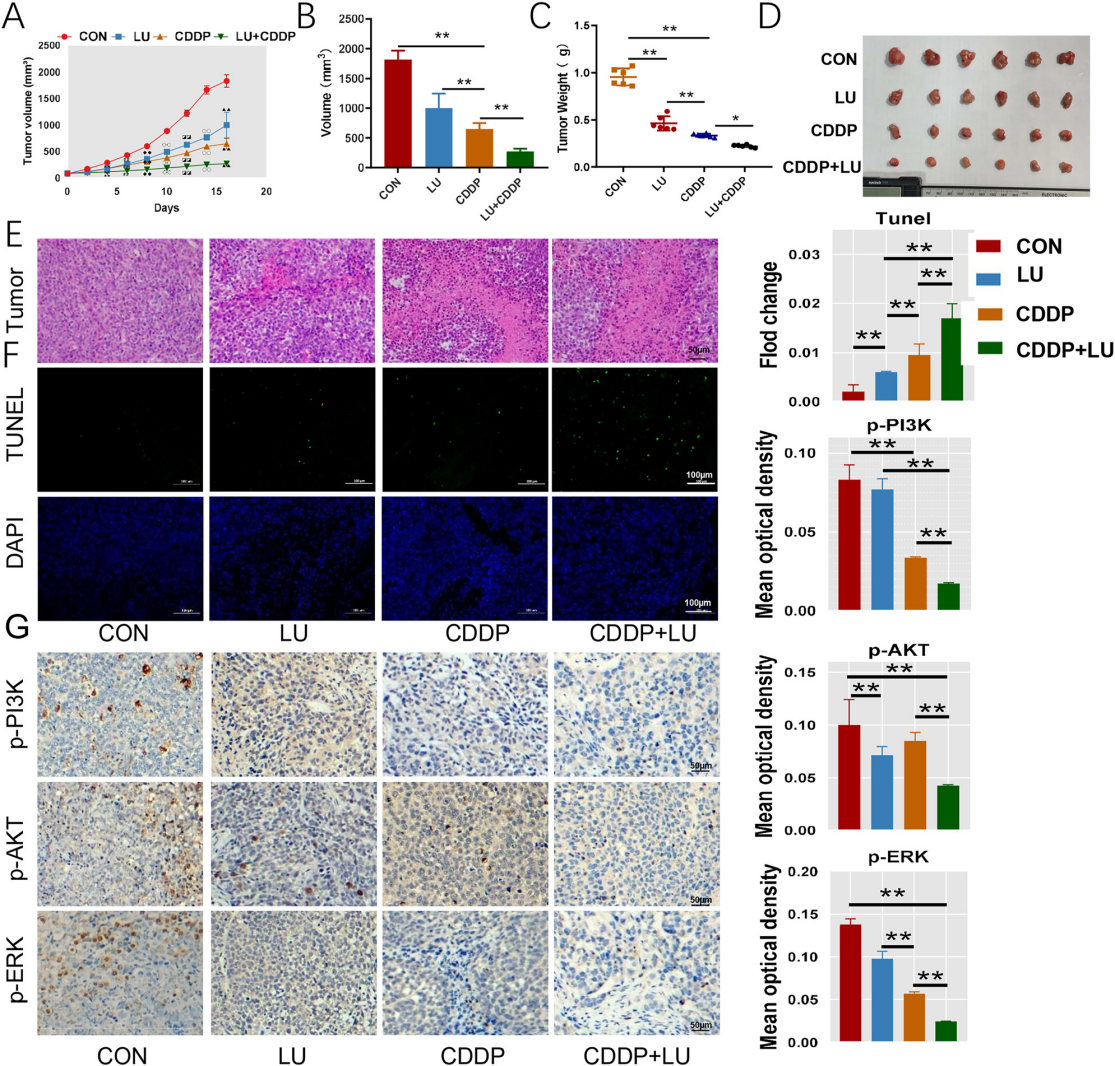
LU enhanced the antitumor effect of CDDP *in vivo*. A, B, C and D. Volume growth curve of tumours in tumour-bearing mice, tumour volume and weight, and photographs of tumour. E. Haematoxylin and eosin (H&E) staining of the histological changes in the deparaffinized sections of mouse tumour tissues. F. Deparaffinized sections of mouse tumour tissues were stained with TUNEL assay. G. Immunohistochemical staining and statistical analysis of the cell proliferation markers p-PI3K, *p*-AKT and *p*-ERK. *P < 0.05, **P < 0.01 compared with control cells. #*P* < 0.05, ##*P* < 0.01 compared with control cells. ◆*P* < 0.05, ◆◆*P* < 0.01 compared with control cells. 〇*P* < 0.05, 〇〇*P* < 0.01 compared with control cells. ◪*P* < 0.05, ◪◪*P* < 0.01 compared with control cells. ◌*P* < 0.05, ◌◌*P* < 0.01 compared with control cells. ▲*P* < 0.05, ▲▲*P* < 0.01 compared with control cells.

**Figure 7:**
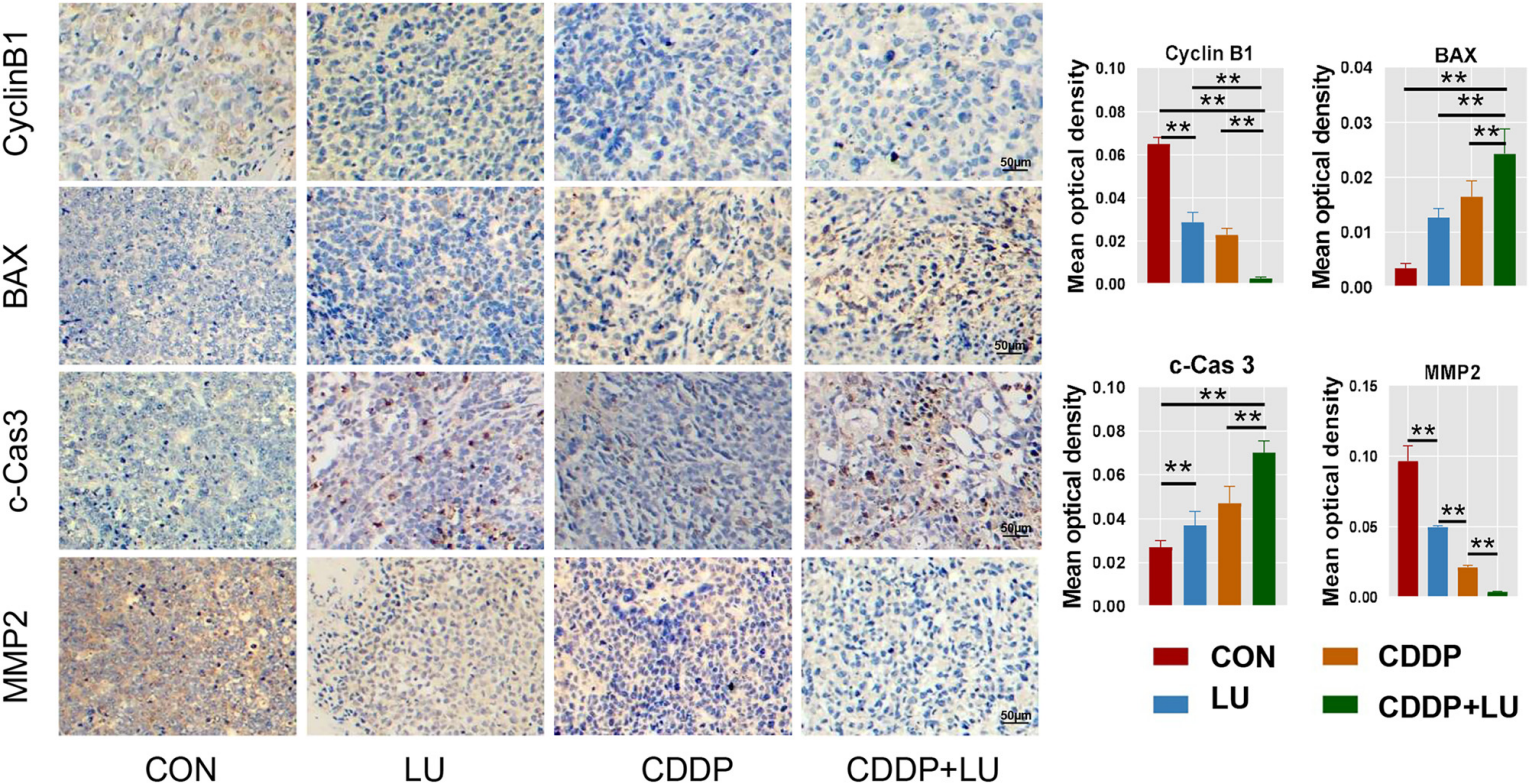
Immunohistochemical staining and statistical analysis of cyclin B1, BAX, c-cas 3 and MMP-2. *P < 0.05, **P < 0.01 compared with control cells.

Tumour-bearing nude mouse models were constructed with HeLa cells to study the synergistic antitumour effect of LU on CDDP *in vivo*. Tumour volume analysis (Figure 6A) showed that the combination group significantly inhibited tumour growth in mice compared with the LU group and the CDDP group (*P* < 0.05). All quantitative statements regarding tumor volume and weight were explicitly reported as mean ± standard deviation (Figure 6B–D). Furthermore,the CDDP group exhibited suppressed tumour weight (*P* < 0.05) compared to the control group (Figure 6C). The inhibitory effect was significantly enhanced by the combination group compared with the CDDP group (*P* < 0.05). In H&E staining of tumour tissue across all treatment groups, the combination therapy group exhibited cellur necrosis structures and nuclear dissolution. No such phenomena were observed in the control group (Figure 6E). To further verify that LU combined with CDDP increases cell apoptosis, TUNEL staining of tumour tissue showed that the number of apoptotic cells observed under a fluorescence microscope was higher in the CDDP group than in the control group, and the green fluorescence apoptotic cells in the combined group were the most abundant (Figure 6F). Immunohistochemical staining was used to detect the cell proliferation markers *p*-PI3K, *p*-AKT and *p*-ERK, the cell cycle markers Cyclin B1, apoptosis markers BAX and c-Cas 3 and the cell migration marker MMP-2 expression. In HeLa tumour-bearing mice, the combination group had the lowest expression of *p*-PI3K, *p*-AKT, *p*-ERK, Cyclin B1 and MMP2 and the highest expression of BAX and c-Cas3 compared (*P* < 0.05) with the control group and the LU and CDDP groups (Figure 6G and 7). Immunohistochemical results of tumour tissue were consistent with the results of protein expression levels in HeLa cells after *in vitro* treatment.

**Figure 8:**
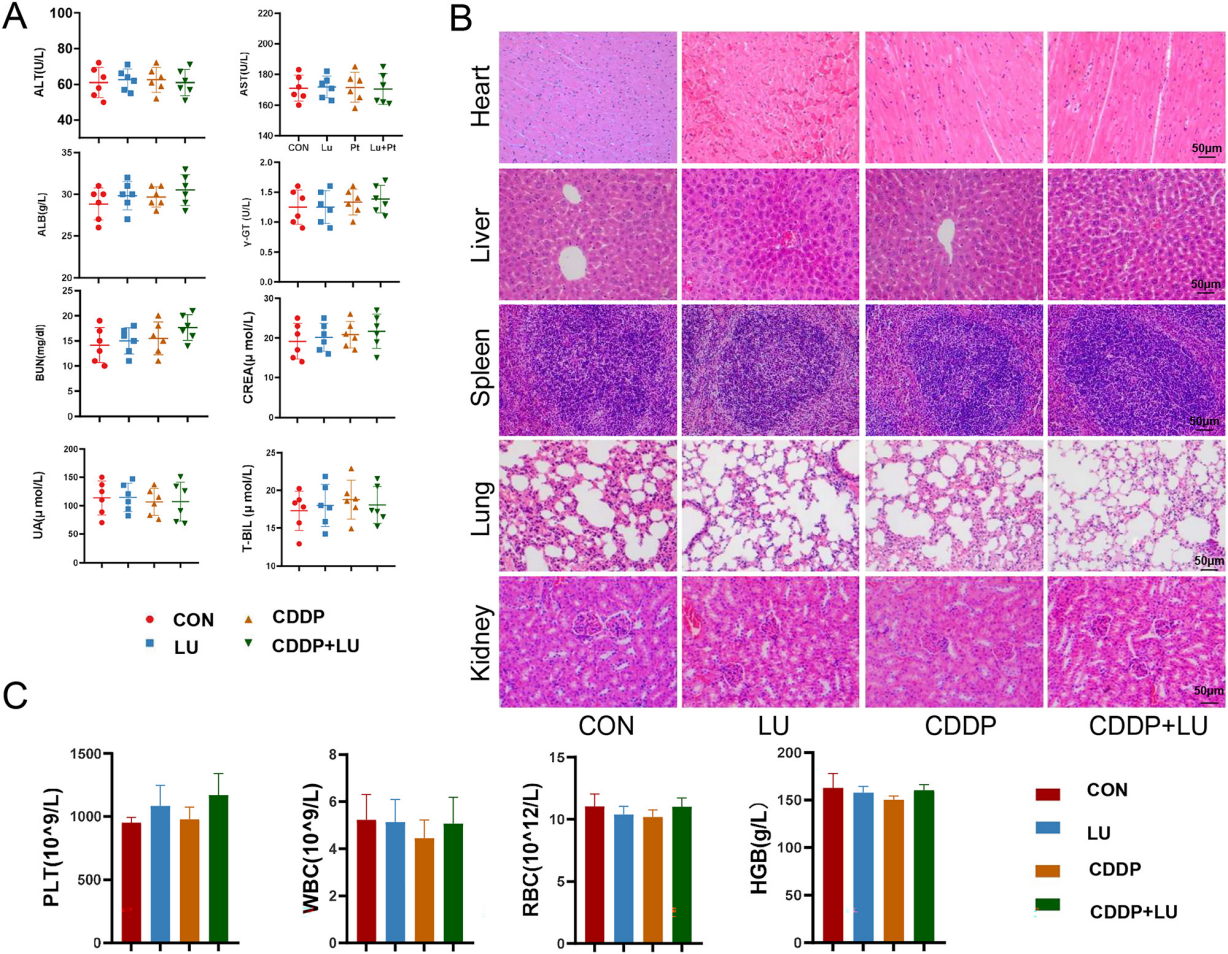
Biosafety assessments of LU and CDDP treatments. A. Liver function index and renal function index examinations of whole blood samples, including alanine transaminase (ALT) count, aspartate aminotransferase (AST) count, albumin (ALB) count, *γ*-glutamyl transpeptidase (γ-GT) count, total bile acid (t-BIL) count, blood urea nitrogen (BUN) count, creatinine (CREA) count, and uric acid (UA) count. B. Haematoxylin and eosin staining of the histological changes in the heart, liver, spleen, lung and kidney. C. Routine blood examinations of white blood cell (WBC) count, red blood cell (RBC) count, haemoglobin (HGB) count and blood platelet (PLT) count.

The H&E staining results of heart, liver, spleen, lung, and kidney tissues revealed intact structures in all treatment groups (Figure 8B). Biochemical parameters, including liver function indices ALT, AST, ALB, *⋎*-GT and T-BIL and kidney function indices BUN, CREA and UA were tested (Figure 8A). The rest of the biochemical indices showed no difference among the treatment groups and the control group. Routine blood indices WBC, RBC, PLT and HGB were tested (Figure 8C), and no significant difference was found in routine blood indices between each treatment group and the control group.

## 4 Discussion

In this study, network analysis results showed that LU, an active component of BJ, synergistically enhances the antitumor effect of CDDP against cervical cancer, potentially through modulation of the PI3K/AKT signaling pathway [19], 20]. Considering that the PI3K/AKT pathway affects the cell cycle, apoptosis and metastasis of tumours, *in vitro* experiments of these three aspects were carried out separately to verify the mechanism of LU sensitization to CDDP. Finally, the abovementioned effects were verified *in vivo* in nude mouse tumour-bearing experiments, including the inhibition of tumour growth, apoptosis and the expression of some related proteins.

It has been reported that the cell cycle is closely related to the occurrence and development of tumour [21]. CDDP induces G2 phase arrest in cervical cancer cells [22], and we found that LU effectively enhanced this effect, which was consistent with the role of LU in human colon cancer cells and non-small cell lung cancer cells [21], 23]. Furthermore, the cell cycle was mainly composed of G1/G0 and G2/M phases, coordinated by cyclin/CDK complexes and CDK inhibitors. CDDP regulates G2/M phase through CDC1/CDK2 and a B-type cyclin, and the decreased expression of Cyclin B1 results in a dose-dependent arrest of cells in G2/M phase [21]. This was consistent with the findings of this study and the conditioning effect was significantly enhanced by LU. These results suggested that LU could enhance the G2/M arrest of HeLa cells induced by CDDP, thereby reducing the proliferation of HeLa cells.

Induction of apoptosis is a key part of inhibiting tumour development and is at least partially regulated by caspases and Bcl-2 family proteins. Permeabilization of the mitochondrial outer membrane (MOMP), caused by oligomerization of proapoptotic effector proteins to apoptotic stressors, releases cytochrome *c* to cleave caspase-3 [24]. Activated caspase 3 binds to apoptotic protease activating factor-1 (Apaf-1) and caspase 9 in the context of ATP, causing irreversible cell death. CDDP can promote the apoptosis of various tumour cells, including melanoma cells and colorectal cancer cells, by upregulating c-caspase-3 and BAX/BCL-2 [25], 26]. This study demonstrated that LU can synergize with CDDP to induce apoptosis of cervical cancer HeLa cells both *in vivo* and *in vitro*.

Tumour metastasis is related to epithelial-mesenchymal transition (EMT), and it is a related factor for the death of tumour patients [27]. The extracellular matrix is modified and degraded by matrix metalloproteinases (MMPs), which are activated by EMT-activated cells, leading to cell detachment [27]. In this study, the ability of CDDP to inhibit the invasion and migration of HeLa cells was verified, which is consistent with the effect of CDDP on MCF-7 cancer cells [27]. LU also has the ability to inhibit tumour cell metastasis in androgen receptor-positive triple-negative breast cancer (TNBC) cells [28].

It has been verified that LU can block the signal transduction of the PI3K/AKT pathway to inhibit the occurrence and development of various cancers, such as melanoma and tamoxifen-resistant breast cance^r^ [29], 30]. Our network analysis results suggest that LU also exerts anticancer effects on cervical cancer through the PI3K/AKT pathway. The PI3K pathway provides essential metabolites that promote tumour cell growth through glycolysis and lipogenesis and controls angiogenesis, proliferation and growth through mechanisms such as vascular endothelial growth factor (VEGF) transcriptional activation. This demonstrates the importance of PI3K signalling and its significant contribution to cellular transformation and cancer development. In addition, inhibition of PI3K/AKT activation leads to downregulation of Cyclin B1 accumulation, thereby preventing cell progression from G2 to mitosis [31]. Our results showed that in a tumour-bearing nude mouse model treated with LU and CDDP, immunohistochemical results showed marked inactivation of the signalling cascades of PI3K, *P*-PI3K, AKT and P-AKT, thereby inhibiting tumour proliferation. In addition, overactivation of ERK in the MAPK pathway is another mechanism of tumour cell resistance to chemotherapeutic drugs. The mitogenic effect in tumour cells is affected by the level of MAPK/ERK. ERK, as s key mediator, allows growth factors to achieve their mitotic potential [32]. In the *in vivo* nude mouse transplantation tumor model of this study, immunohistochemistry results showed that both LU and CDDP reduced the expression level of *P*-ERK, with the most significant reduction in the combined treatment group. Based on the above results and biosafety testing, LU may represent a safe, effective, and promising platinum sensitizer capable of synergistically enhancing platinum sensitivity in HeLa cells to CDDP.

However, there are several limitations to this study. First, the information from the online database is based on reviewed and predicted data; thus, compounds or targets that are unproven and undocumented may not be included in our analysis. Second, currently, the quantitative determination of BJ’s 15 compounds is not complete; thus, content determination should be carried out in the future. Third, further mechanistic studies using pathway inhibitors or gene knockout techniques will strengthen the causal relationship between predicted pathways and observed phenotypes. In addition, LU, while identified as the most important bioactive ingredient in BJ against CC, is not fully representative of BJ. Therefore, further research is needed to explore the potential molecular mechanisms of BJ *in vitro* and *in vivo* for the treatment of CC.

## 5 Conclusions

In conclusion, this study employed a network analysis approach to analyse the active components and potential targets of BJ and LU against CC. Furthermore, it was verified that LU could inhibit cell proliferation by inhibiting the PI3K/Akt signalling pathway and downregulating ERK expression, synergistically enhancing the anticancer activity of CDDP against CC without increasing side effects. LU may be the main active ingredient of BJ anti-CC. This suggests that BJ is a promising platinum sensitizer.

## References

[1] Sung H, Ferlay J, Siegel RL, Laversanne M, Soerjomataram I, Jemal A (2021). Global cancer statistics 2020: GLOBOCAN estimates of incidence and mortality worldwide for 36 cancers in 185 countries. CA Cancer J Clin.

[2] Koh WJ, Abu-Rustum NR, Bean S, Bradley K, Campos SM, Cho KR (2019). Cervical cancer, version 3.2019, NCCN clinical practice guidelines in oncology. J Natl Compr Cancer Netw.

[3] Wen X, Liu S, Sheng J, Cui M (2020). Recent advances in the contribution of noncoding RNAs to cisplatin resistance in cervical cancer. PeerJ.

[4] Gu J, Li Z, Zhou J, Sun Z, Bai C (2019). Response prediction to oxaliplatin plus 5-fluorouracil chemotherapy in patients with colorectal cancer using a four-protein immunohistochemical model. Oncol Lett.

[5] Li KW, Liang YY, Wang Q, Li Y, Zhou SJ, Wei HC (2021). Brucea javanica: a review on anticancer of its pharmacological properties and clinical researches. Phytomedicine.

[6] Gao H, Lamusta J, Zhang WF, Salmonsen R, Liu Y, O’Connell E (2011). Tumor cell selective cytotoxicity and apoptosis induction by an herbal preparation from Brucea javanica. N Am J Med Sci.

[7] Ryu S, Park S, Lim W, Song G (2019). Effects of luteolin on canine osteosarcoma: suppression of cell proliferation and synergy with cisplatin. J Cell Physiol.

[8] Chian S, Thapa R, Chi Z, Wang XJ, Tang X (2014). Luteolin inhibits the Nrf2 signaling pathway and tumor growth in vivo. Biochem Biophys Res Commun.

[9] Wang H, Luo Y, Qiao T, Wu Z, Huang Z (2018). Luteolin sensitizes the antitumor effect of cisplatin in drug-resistant ovarian cancer via induction of apoptosis and inhibition of cell migration and invasion. J Ovarian Res.

[10] Wang X, Wang ZY, Zheng JH, Li S (2021). TCM network pharmacology: a new trend towards combining computational, experimental and clinical approaches. Chin J Nat Med.

[11] Zhou Z, Chen B, Chen S, Lin M, Chen Y, Jin S (2020). Applications of network pharmacology in traditional Chinese medicine research. Evid Based Complement Alternat Med.

[12] Zhang R, Zhu X, Bai H, Ning K (2019). Network pharmacology databases for traditional Chinese medicine: review and assessment. Front Pharmacol.

[13] He R, Ou S, Chen S, Ding S (2020). Network pharmacology-based study on the molecular biological mechanism of action for compound kushen injection in anti-cancer effect. Med Sci Monit.

[14] Huang J, Cheung F, Tan HY, Hong M, Wang N, Yang J (2017). Identification of the active compounds and significant pathways of yinchenhao decoction based on network pharmacology. Mol Med Rep.

[15] Liu YY, Yu LH, Zhang J, Xie DJ, Zhang XX, Yu JM (2021). Network pharmacology-based and molecular docking-based analysis of suanzaoren decoction for the treatment of Parkinson’s disease with sleep disorder. BioMed Res Int.

[16] Huang da W, Sherman BT, Lempicki RA (2009). Systematic and integrative analysis of large gene lists using DAVID bioinformatics resources. Nat Protoc.

[17] Wu S, Zhao F, Zhao J, Li H, Chen J, Xia Y (2019). Dioscin improves postmenopausal osteoporosis through inducing bone formation and inhibiting apoptosis in ovariectomized rats. Biosci Trends.

[18] Venkatraman M, Anto RJ, Nair A, Varghese M, Karunagaran D (2005). Biological and chemical inhibitors of NF-kappaB sensitize SiHa cells to cisplatin-induced apoptosis. Mol Carcinog.

[19] Yu M, Qi B, Xiaoxiang W, Xu J, Liu X (2017). Baicalein increases cisplatin sensitivity of A549 lung adenocarcinoma cells via PI3K/Akt/NF-κB pathway. Biomed Pharmacother.

[20] Li X, Zhao J, Yan T, Mu J, Lin Y, Chen J (2021). Cyanidin-3-O-glucoside and cisplatin inhibit proliferation and downregulate the PI3K/AKT/mTOR pathway in cervical cancer cells. J Food Sci.

[21] Chen Z, Zhang B, Gao F, Shi R (2018). Modulation of G(2)/M cell cycle arrest and apoptosis by luteolin in human colon cancer cells and xenografts. Oncol Lett.

[22] Pérez-Rojas JM, González-Macías R, González-Cortes J, Jurado R, Pedraza-Chaverri J, García-López P (2016). Synergic effect of α-Mangostin on the cytotoxicity of cisplatin in a cervical cancer model. Oxid Med Cell Longev.

[23] Cai X, Ye T, Liu C, Lu W, Lu M, Zhang J (2011). Luteolin induced G2 phase cell cycle arrest and apoptosis on non-small cell lung cancer cells. Toxicol Vitro.

[24] Yuan Z, Dewson G, Czabotar PE, Birkinshaw RW (2021). VDAC2 and the BCL-2 family of proteins. Biochem Soc Trans.

[25] Chen HM, Lai ZQ, Liao HJ, Xie JH, Xian YF, Chen YL (2018). Synergistic antitumor effect of brusatol combined with cisplatin on colorectal cancer cells. Int J Mol Med.

[26] Shibuya H, Kato Y, Saito M, Isobe T, Tsuboi R, Koga M (2003). Induction of apoptosis and/or necrosis following exposure to antitumour agents in a melanoma cell line, probably through modulation of Bcl-2 family proteins. Melanoma Res.

[27] Song XQ, Ma ZY, Wu YG, Dai ML, Wang DB, Xu JY (2019). New NSAID-Pt(IV) prodrugs to suppress metastasis and invasion of tumor cells and enhance anti-tumor effect in vitro and *in vivo*. Eur J Med Chem.

[28] Wu HT, Lin J, Liu YE, Chen HF, Hsu KW, Lin SH (2021). Luteolin suppresses androgen receptor-positive triple-negative breast cancer cell proliferation and metastasis by epigenetic regulation of MMP9 expression via the AKT/mTOR signaling pathway. Phytomedicine.

[29] Yao X, Jiang W, Yu D, Yan Z (2019). Luteolin inhibits proliferation and induces apoptosis of human melanoma cells in vivo and in vitro by suppressing MMP-2 and MMP-9 through the PI3K/AKT pathway. Food Funct.

[30] Wu HT, Liu YE, Hsu KW, Wang YF, Chan YC, Chen Y (2020). MLL3 induced by luteolin causes apoptosis in tamoxifen-resistant breast cancer cells through H3K4 monomethylation and suppression of the PI3K/AKT/mTOR pathway. Am J Chin Med.

[31] Liu M, Qi Z, Liu B, Ren Y, Li H, Yang G (2015). RY-2f, an isoflavone analog, overcomes cisplatin resistance to inhibit ovarian tumorigenesis via targeting the PI3K/AKT/mTOR signaling pathway. Oncotarget.

[32] Fang J, Zhou Q, Shi XL, Jiang BH (2007). Luteolin inhibits insulin-like growth factor 1 receptor signaling in prostate cancer cells. Carcinogenesis.

